# Supplementary material for: Gating intermediates reveal inhibitory role of the voltage sensor in a cyclic nucleotide-modulated ion channel
Source: Nat Commun. 2022 Nov 14;13:6919. doi: 10.1038/s41467-022-34673-z (PMC9663499; doi:10.1038/s41467-022-34673-z)
Supplement: Supplementary file 1 — Supplementary Information [file 41467_2022_34673_MOESM1_ESM.pdf]

# Gating intermediates reveal inhibitory role of the voltage sensor in a cyclic nucleotide-modulated ion channel

Xiaolong Gao[1,*], Philipp A.M. Schmidpeter[1,*], Vladimir Berka[2], Ryan J. Durham[2], Chen Fan[1,$], Vasanthi Jayaraman[2], Crina M. Nimigean[1,3,#]

[1] Department of Anesthesiology, Weill Cornell Medical College, 1300 York Avenue, New York, NY 10065

[2] Department of Biochemistry and Molecular Biology, University of Texas Health Science Center, Houston, TX, United States

[3] Department of Physiology and Biophysics, Weill Cornell Medical College, 1300 York Avenue, New York, NY 10065

[$] current address: Science for Life Laboratory, Department of Biochemistry and Biophysics, Stockholm University, Solna, Sweden

* Equal contribution

[#] Correspondence: crn2002@med.cornell.edu

## Supplementary Information

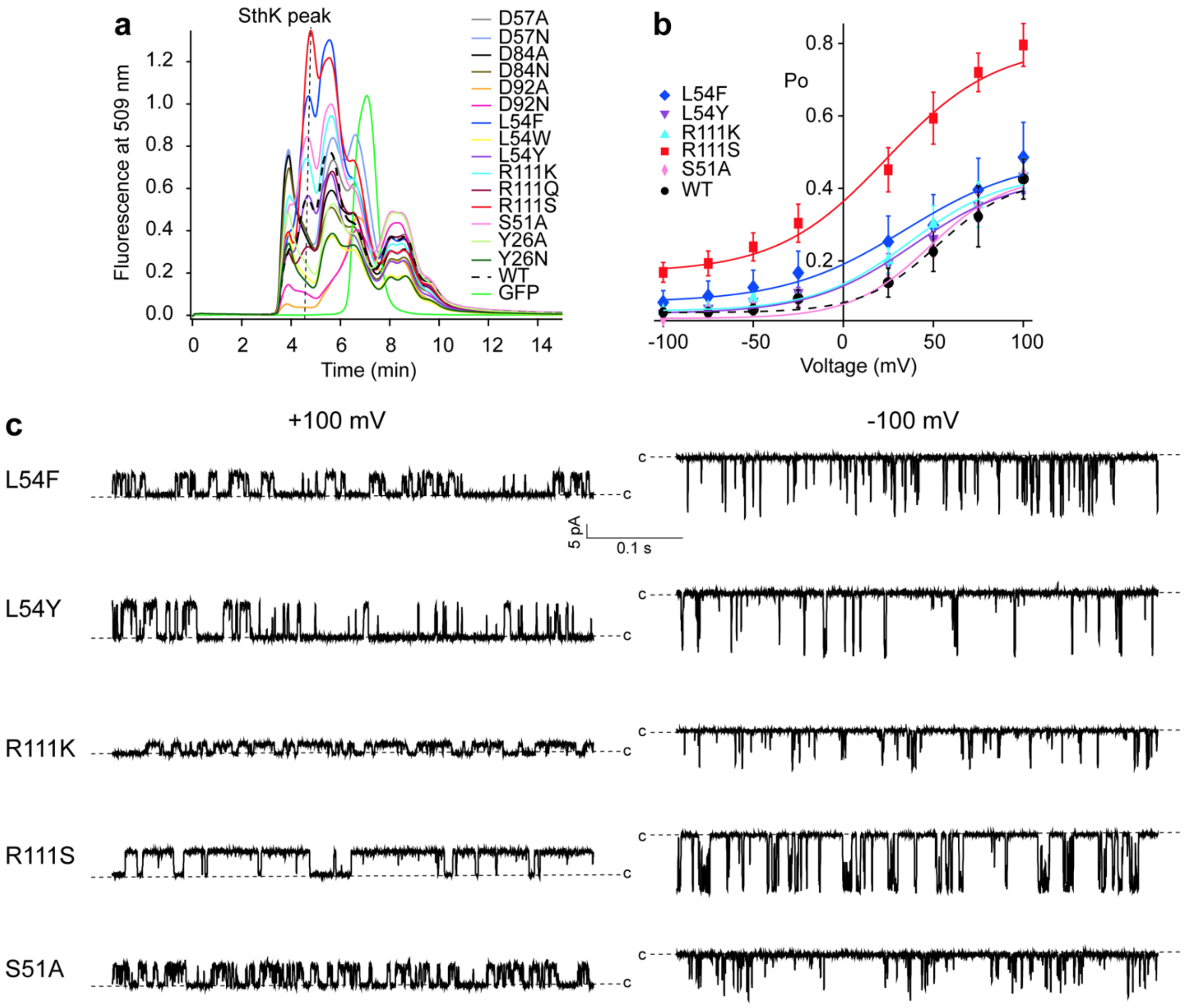


**Supplementary Fig. 1: Expression and functional screening of SthK mutants**

**a** Fluorescence detection size exclusion chromatography (FSEC, Superdex200 5/150) profiles for all VSD mutant SthK channels generated to screen for protein expression. Green fluorescence protein (GFP, green line) and WT SthK (dashed line) are shown as reference. **b** Po/voltage plot from single channel recordings of SthK mutants that showed a peak for tetrameric protein in the FSEC screen (SthK S51A, L54F, L54Y, R111K, and R111S with colors as in **a**). Lines are fits according to Eq 1, numerical values are listed in Supplementary Table 1. n > 4 independent experiments for all data points. SthK R111S yielded unstable channel protein, showed a strongly shifted $V_{half}$ as compared to WT SthK, and expressed poorly in large scale cultures and hence was not further analyzed. **c** Single channel recordings of mutant SthK channels shown in **b** at +100 mV (left column) and -100 mV (right column) in the presence of 200 μM cAMP. The mutations and the closed levels (dashed lines) are indicated.

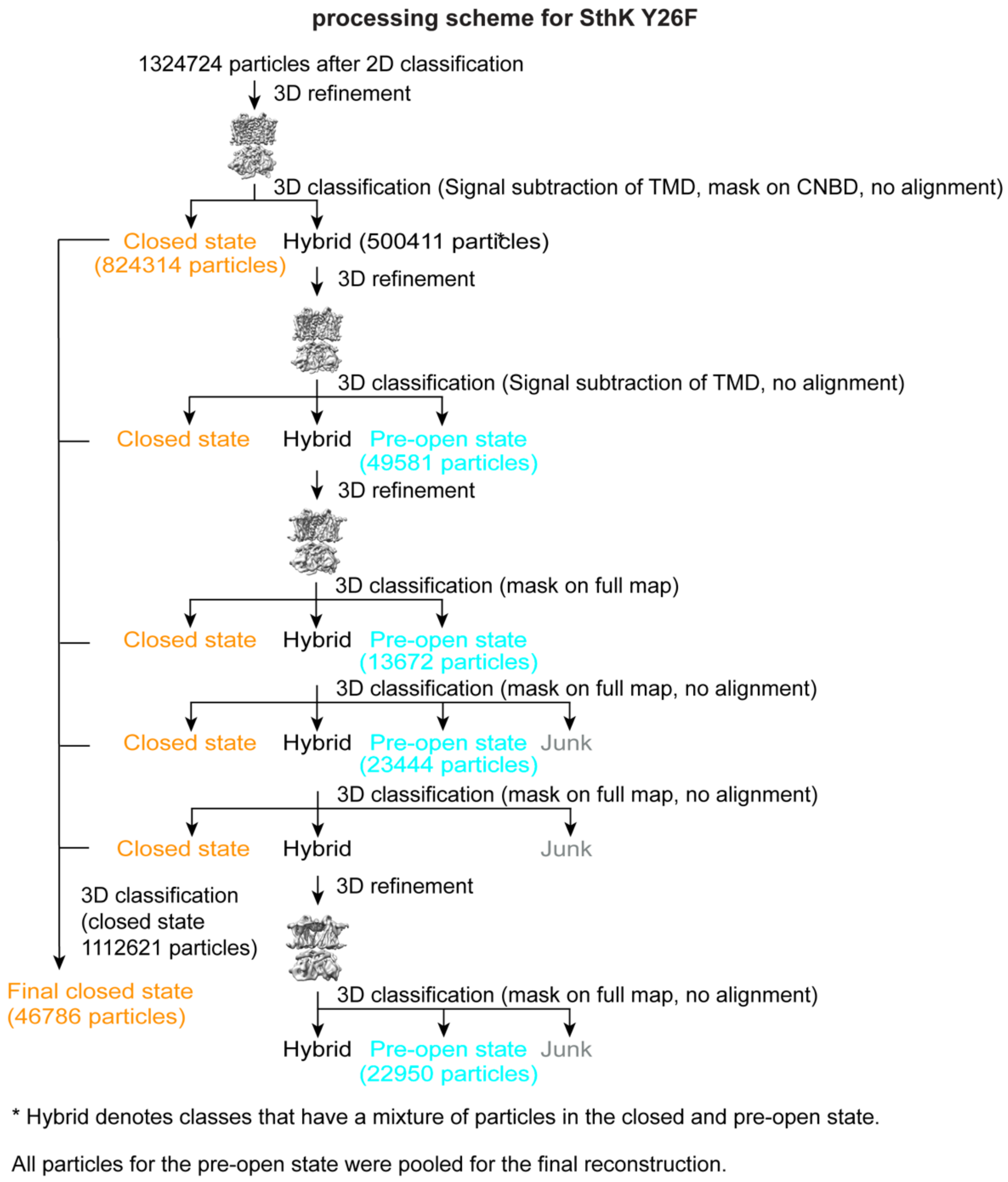


**Supplementary Fig. 2: Cryo-EM data processing scheme for SthK Y26F**
The main processing steps employed to separate particles for the closed state and the activated, pre-open state of SthK Y26F are summarized in a flow chart. Particle numbers in key classes are indicated.

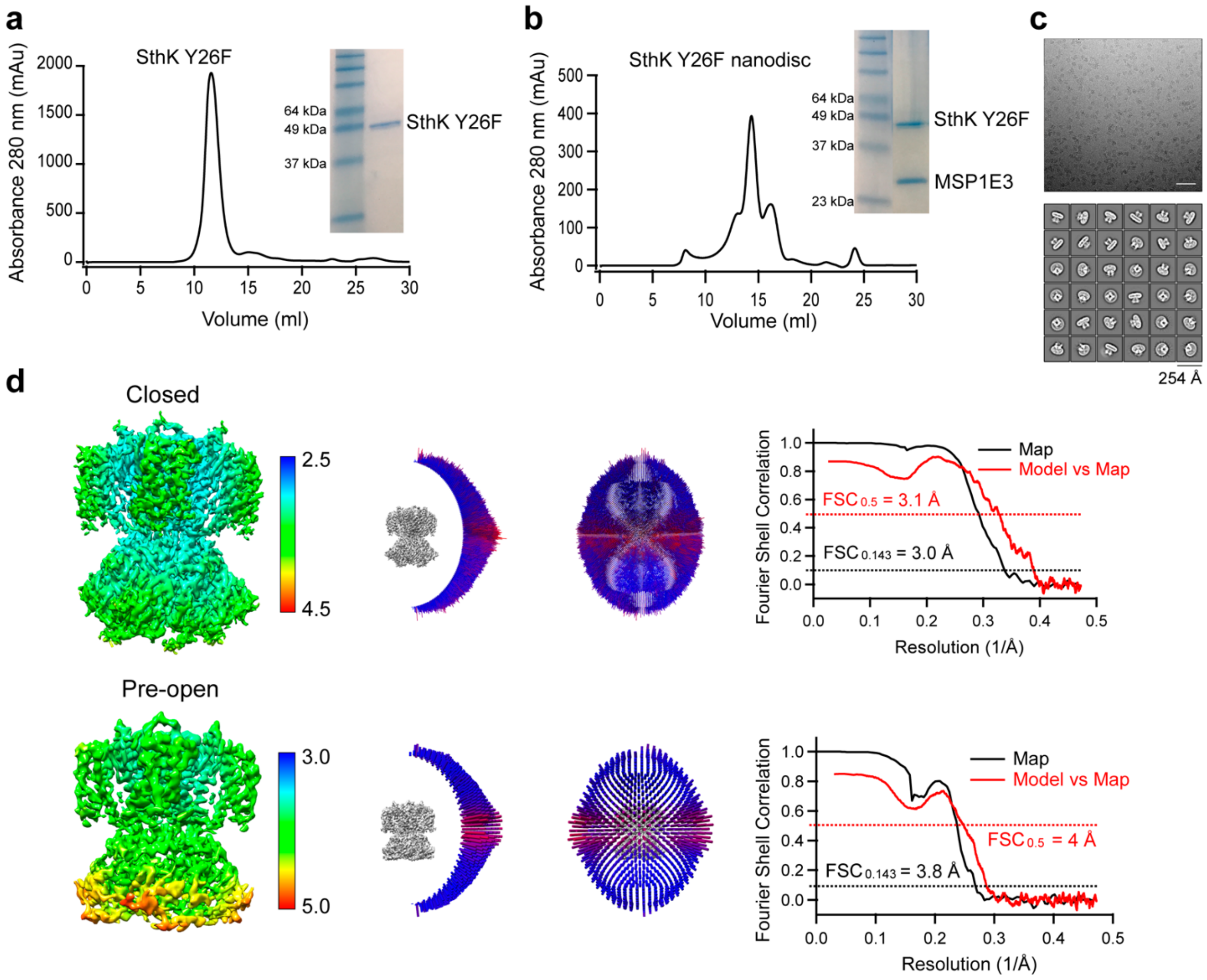


**Supplementary Fig. 3: Sample preparation and Cryo-EM structure validation of SthK Y26F**
**a** Gel filtration profile (Superdex200 10/300) of SthK Y26F in detergent and SDS-PAGE of the final sample (BenchMark$^{TM}$ Pre-Stained Protein Standard, ThermoFisher). **b** Gel filtration (Superose6 10/300) of SthK Y26F reconstituted into nanodiscs and SDS-PAGE (BenchMark$^{TM}$ Pre-Stained Protein Standard, ThermoFisher) of final sample used for cryo-EM analysis. **c** Representative micrograph (scale bar 50 nm) and 2D class averages (box size indicated) for the SthK Y26F dataset. **d** Local resolution, angular distribution, and FSC curves for the final cryo-EM density maps and the models are shown (from left to right). Top row: closed state SthK Y26F. Bottom row: activated, pre-open SthK Y26F.

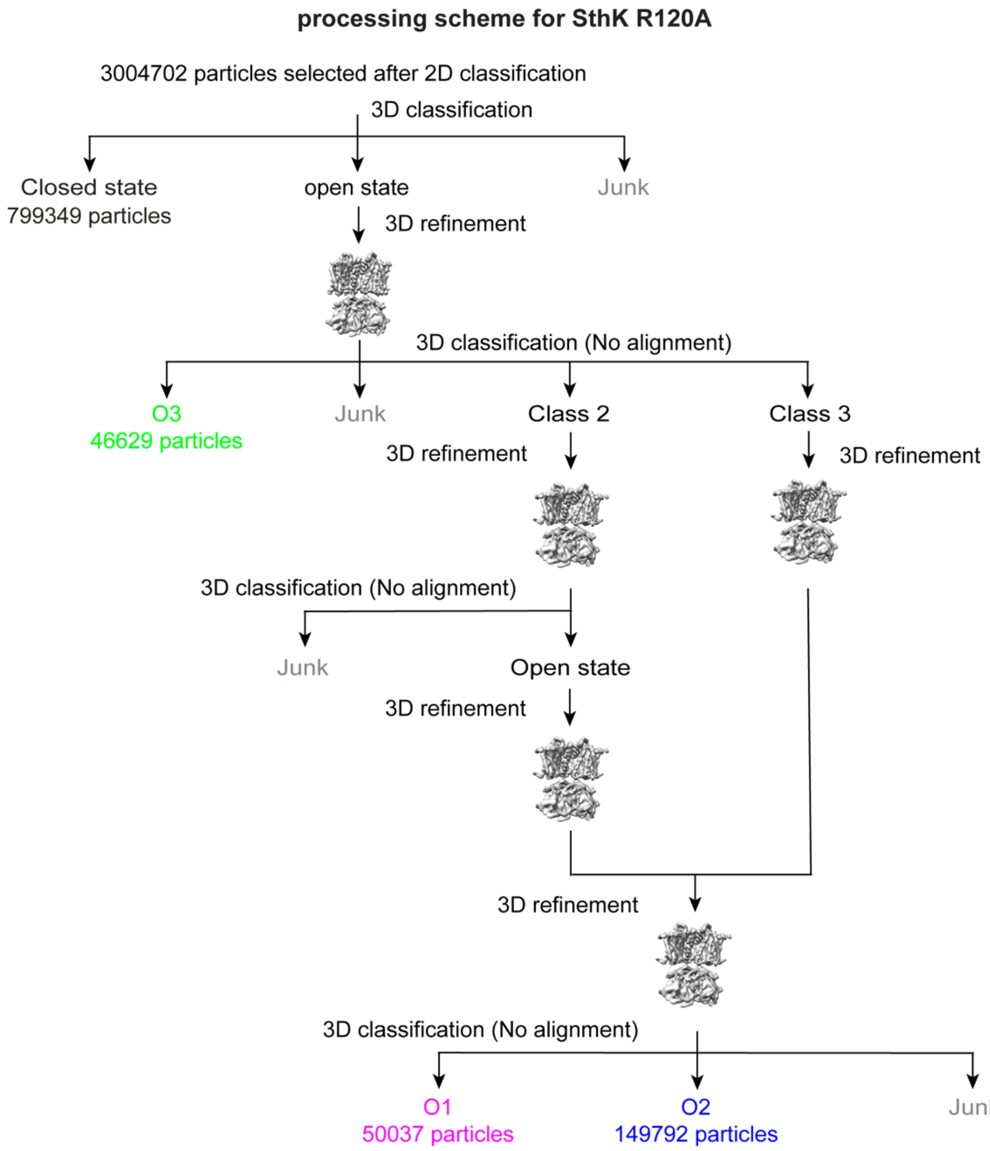


**Supplementary Fig. 4: Cryo-EM data processing scheme for SthK R120A**
The main processing steps employed to separate particles for the closed state and the three different open states (O1, O2, O3) of SthK R120A are given. Particle numbers are indicated for each state.

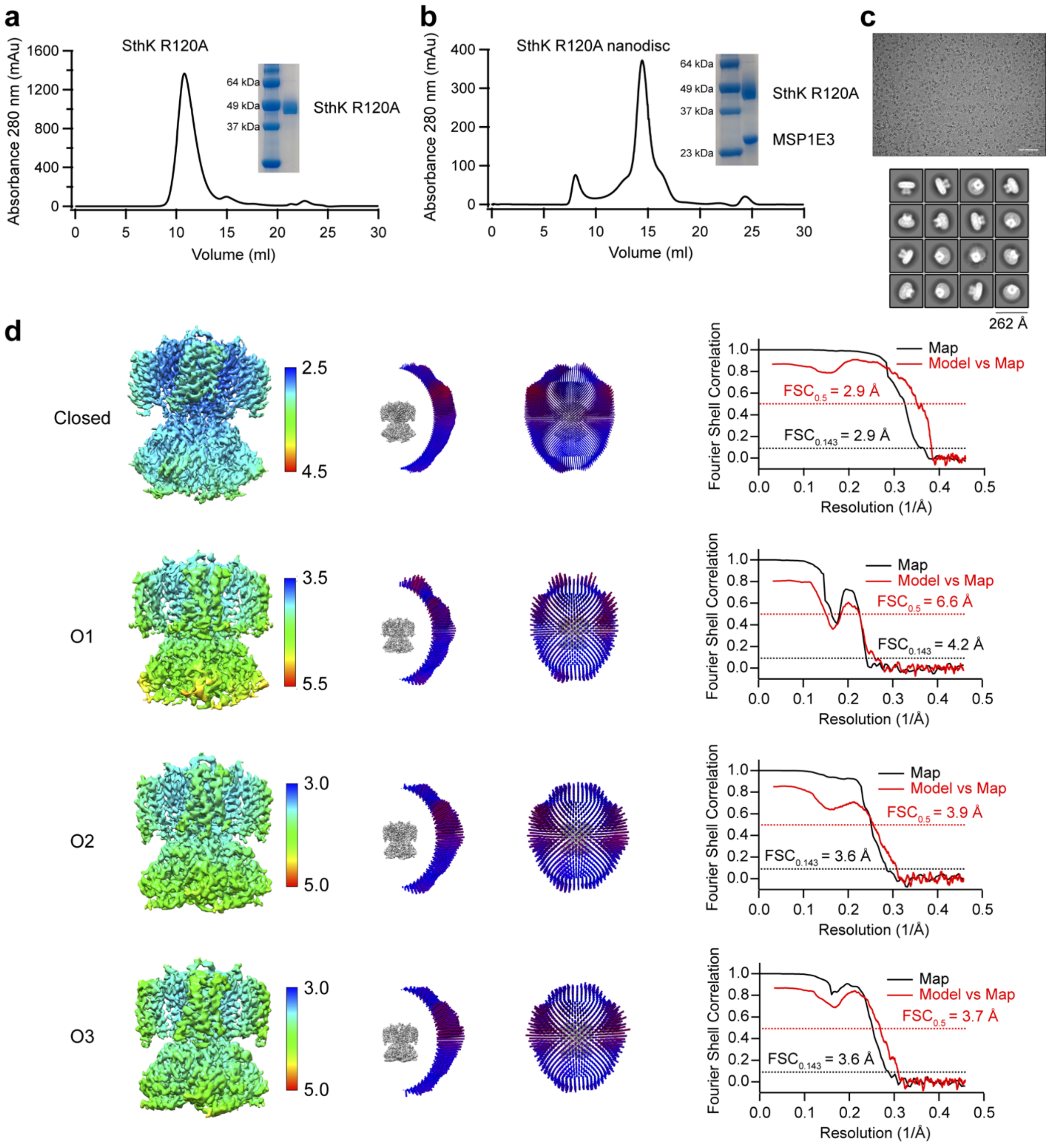


**Supplementary Fig. 5: Sample preparation and structure validation of SthK R120A**

**a** Gel filtration profile (Superdex200 10/300) of SthK R120A in detergent, SDS-PAGE of the final sample (BenchMark$^{TM}$ Pre-Stained Protein Standard, ThermoFisher). **b** Gel filtration (Superose6 10/300) of SthK R120A reconstituted into nanodiscs, SDS-PAGE (BenchMark$^{TM}$ Pre-Stained Protein Standard, ThermoFisher) of final sample used for cryo-EM. **c** Representative micrograph (scale bar 50 nm) and 2D class averages (box size indicated) for SthK R120A. **d** Local resolution, angular distribution, and FSC curves for the final cryo-EM density maps and the models are shown (from left to right). Rows from top to bottom show: closed state, open state 1 (O1), open state 2 (O2), and open state 3 (O3) of SthK R120A.

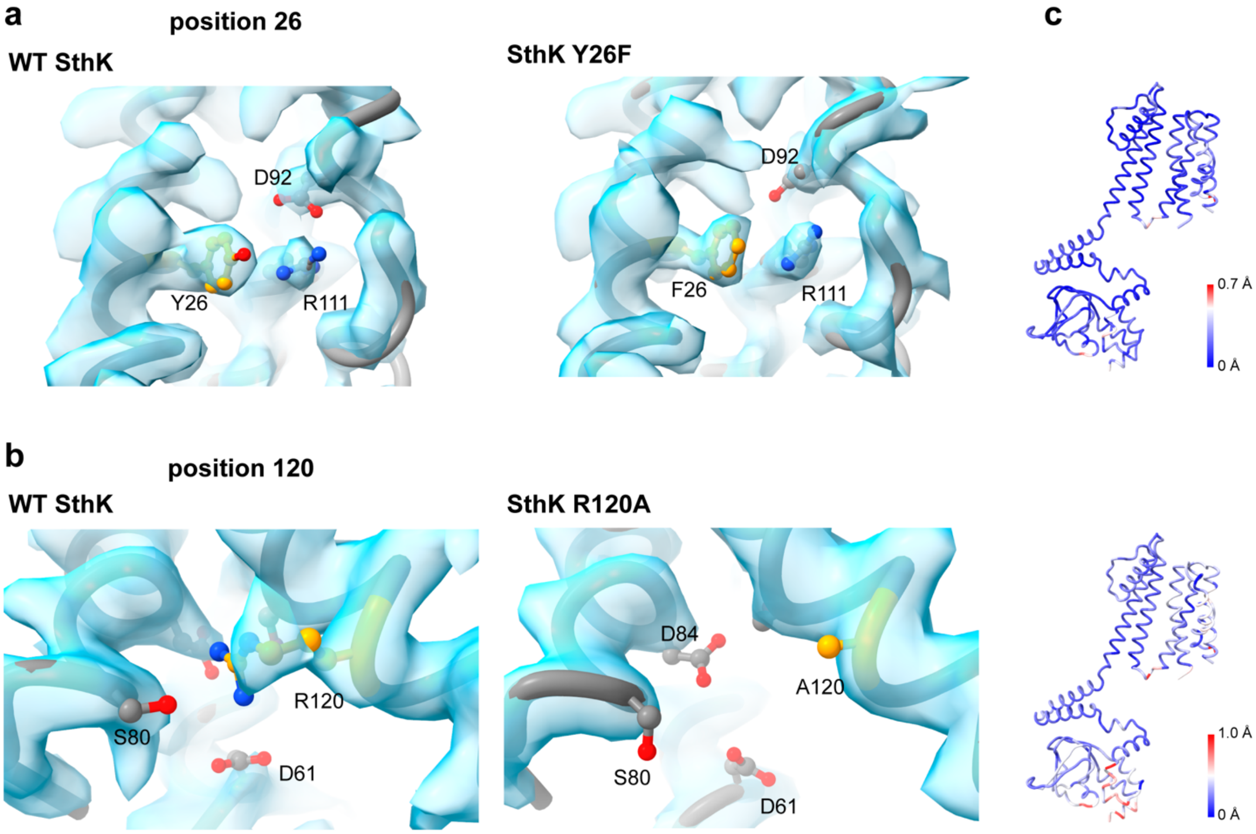


**Supplementary Fig. 6: Mutations Y26F and R120A did not alter the structures of the SthK channels in the closed conformation.**

**a** Details of the cryo-EM map and model around position 26: Y26 (WT SthK, left) and F26 (SthK Y26F, right). **b** Details of the cryo-EM map and model around position 120: R120 (WT SthK, left) and A120 (SthK R120A, right). Protein chain is shown as grey ribbon, select residues interacting with the mutated residues are shown in ball-and-stick representation. Positions 26 and 120 are highlighted in orange. WT SthK panels were prepared from PDB: 6cju and EMDB: 7484 (Rheinberger et al., 2018). **c** To highlight lack of global changes induced by the mutations Y26F and R120A, one subunit of SthK is shown in cartoon representation colored by RMSD between WT SthK and SthK Y26F (top) or WT SthK and SthK R120A (bottom).

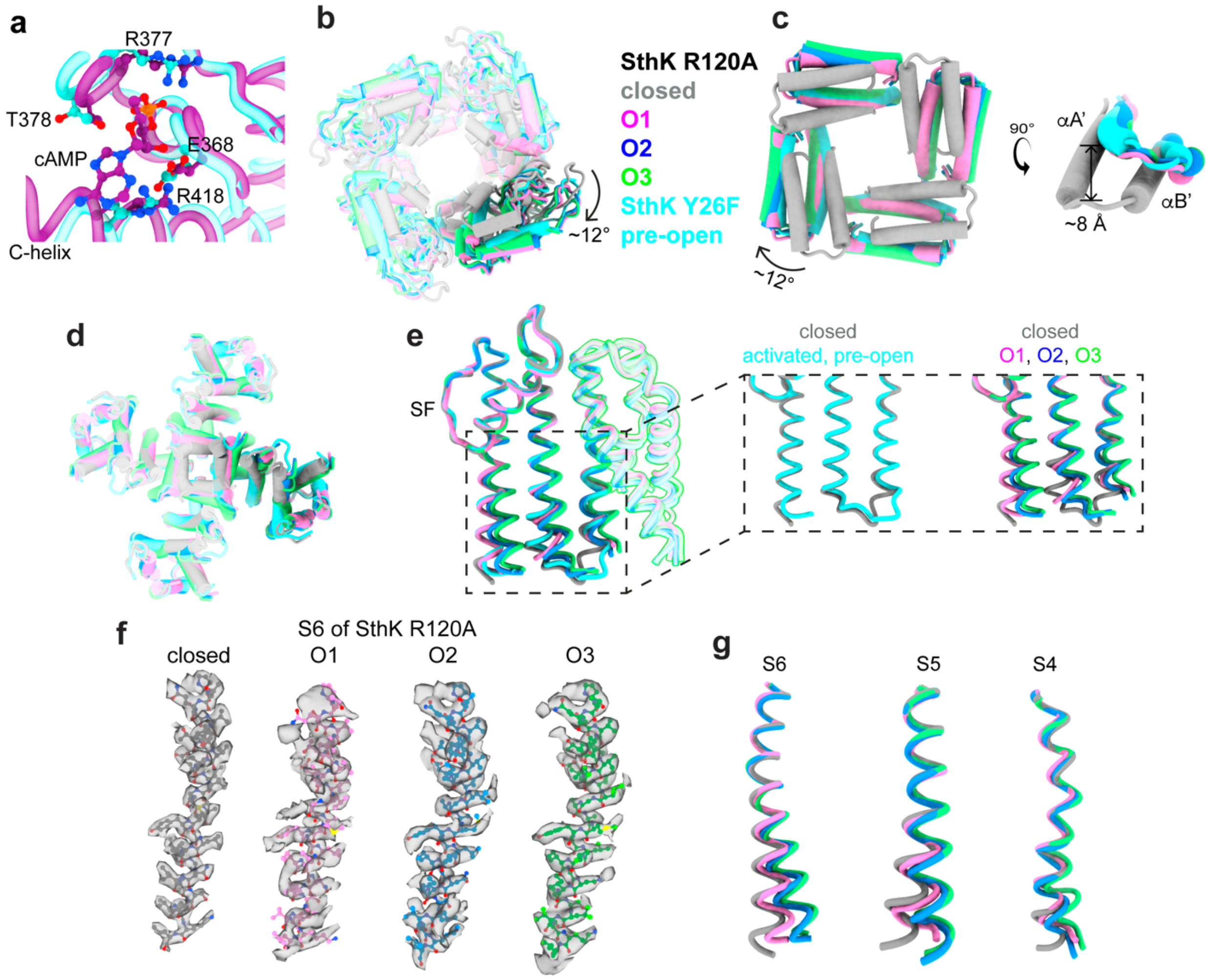


**Supplementary Fig. 7: Structural rearrangements in SthK Y26F and SthK R120A**
**a** Overlay of the cAMP binding site of activated, pre-open SthK Y26F (cyan) and the X-ray crystal structure of the isolated, activated CNBD (violet, PDB: 4d7t). **b** Bottom view of the CNBDs for SthK R120A closed (grey), O1 (pink), O2 (blue), O3 (green), and activated, pre-open SthK Y26F (cyan) showing that the CNBDs of pre-open and open states undergo similar movements. **c** Bottom view of the A' and B' helices of the C-linker with colors as in **b** showing that the C-linkers of pre-open and open states undergo the same movements. **d** Bottom view of the TMDs (colors as in **b**) showing S6 and S5 movements. **e** TMD of one subunit (colors as in **b**) with S1-S3 transparent showing different movements of S4-S6 in pre-open and open states. The inset shows the bottom of S4-S6 separately for the pre-open and the three open states, in comparison to the closed state. **f** Cryo-EM densities and models of the S6 helices for the four different states identified in the SthK R120A dataset showing that the models depict distinct states. Densities are in transparent grey, models are colored as in **b**. **g** Overlay of the isolated S4, S5 and S6 helices of SthK R120A in the four different states (colors as in **b**) showing a gradual outwards movement of all three helices from closed < O1 < O2 < O3.

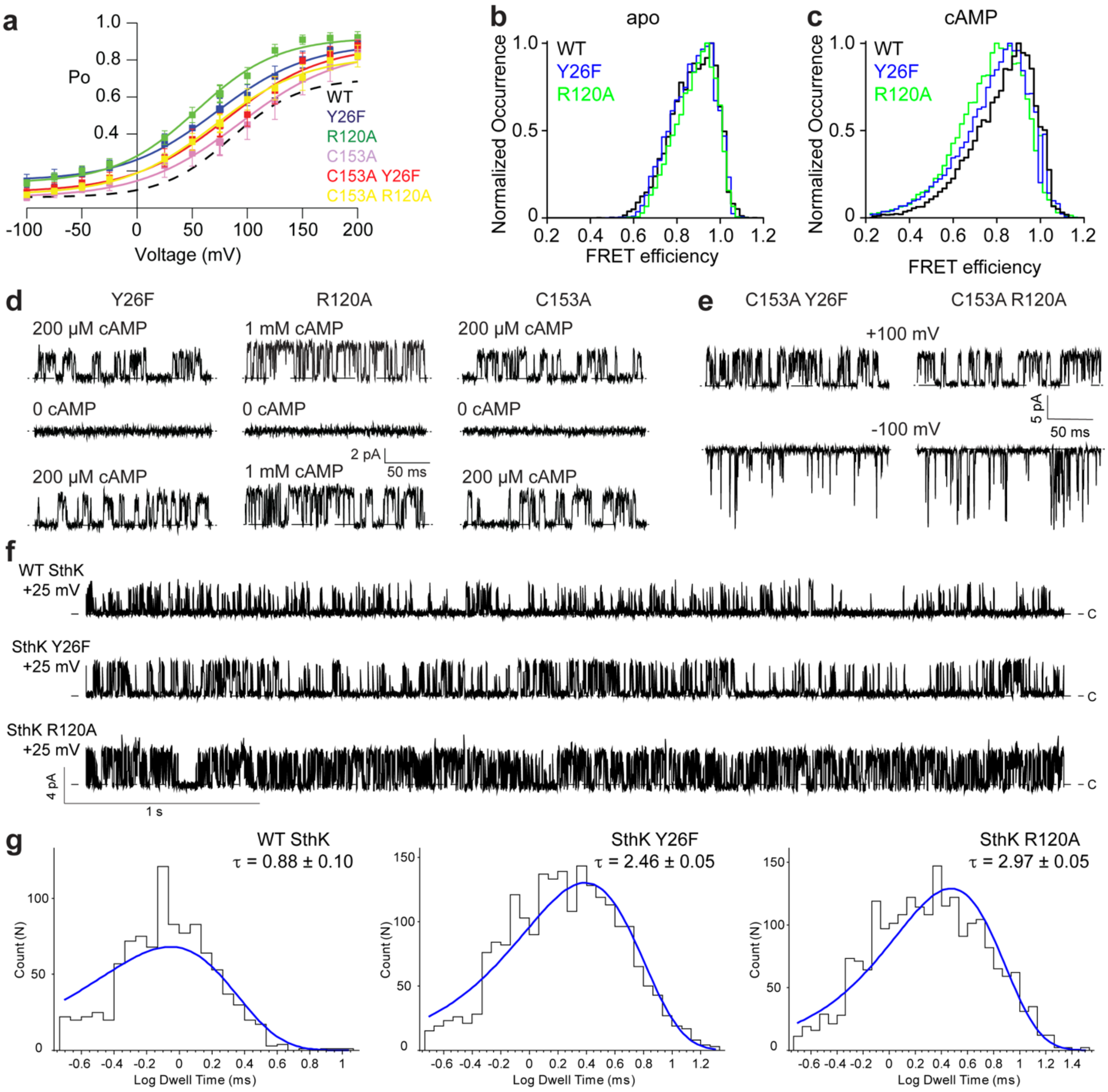


**Supplementary Fig. 8: Single molecule analyses of SthK gating intermediates**

**a** Open probability of SthK channels used for sm-FRET as function of the applied voltage. Numerical values obtained from fits according to Eq 1 are summarized in Supplementary Table 1. **b** and **c** Histograms of FRET efficiency distribution for SthK in the absence **b** and presence **c** of cAMP for WT SthK (black), SthK Y26F (blue), and SthK R120A (green). **d** Representative single channel recordings for SthK Y26F, SthK R120A, SthK C153A at +100 mV in the presence of cAMP, after perfusion of 0 µM cAMP, and after perfusion back to saturating cAMP (from top to bottom) showing reversibility of cAMP activation. **e** Representative single channel recordings of SthK C153A Y26F (left) and SthK C153A R120A (right) at +100 mV (top row) and -100 mV (bottom row) in the presence of 200 µM cAMP showing that the single channel characteristics of these mutants are similar to WT SthK. **f** Single channel recordings of WT SthK, SthK Y26F, SthK R120A (top to bottom) at +25 mV. Closed levels are indicated by dashed lines for all single channel recordings. **g** Open dwell time histograms for WT SthK, SthK Y26F, SthK R120A (at +25 mV) generated from recordings as in **f**. Histograms were analyzed using a single log probability function to determine the mean open dwell time $\tau \pm$ S.E.

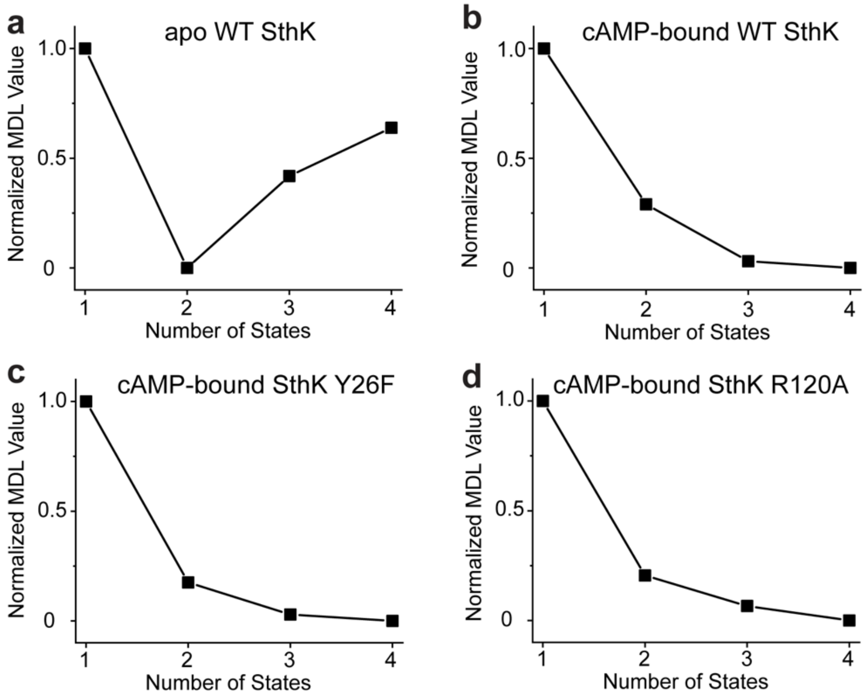


**Supplementary Fig. 9: Minimum Description Length function values for sm-FRET data**
Shown are the values of the Minimum Description Length (MDL) function used to evaluate the number of states in an sm-FRET traces for **a** apo WT SthK, **b** cAMP-bound WT SthK, **c** cAMP-bound SthK Y26F, **d** cAMP-bound SthK R120A. Smaller MDL values indicate a better balance between goodness of the fit to the data and simplicity of the overall model. MDL values were generated by the Step Transition and State Identification (STaSI) software (Shuang et al., 2014). MDL analysis was performed after denoising of the sm-FRET data.

**Supplementary Table 1: Functional parameters of SthK channels**

| SthK protein | Po at -100 mV | n | Po at +200 mV | n | z | $V_{half}$ (mV) |
|---|---|---|---|---|---|---|
| | | | **Voltage dependence** | | | |
| WT # | 0.06 ± 0.01 | 6 | 0.70 ± 0.04 | 4 | 0.8 ± 0.04 | 85 ± 1.7 |
| Y26F | 0.15 ± 0.04 | 12 | 0.89 ± 0.03 | 11 | 0.6 ± 0.02 | 72 ± 2.0 |
| R120A | 0.14 ± 0.02 | 23 | 0.92 ± 0.03 | 4 | 0.7 ± 0.04 | 54 ± 2.5 |
| C153A | 0.06 ± 0.02 | 22 | 0.86 ± 0.06 | 8 | 0.6 ± 0.04 | 93 ± 3.3 |
| C153A Y26F | 0.08 ± 0.02 | 19 | 0.88 ± 0.03 | 7 | 0.6 ± 0.05 | 81 ± 3.7 |
| C153A R120A | 0.07 ± 0.01 | 16 | 0.82 ± 0.06 | 5 | 0.6 ± 0.03 | 70 ± 2.0 |
| | | | **Po at +100 mV** | | | |
| S51A | 0.04 ± 0.01 | 12 | 0.44 ± 0.04 | 6 | 1.2 ± 0.2 | 48 ± 3.6 |
| L54F | 0.09 ± 0.03 | 10 | 0.49 ± 0.10 | 8 | 0.8 ± 0.1 | 34 ± 5.3 |
| L54Y | 0.05 ± 0.01 | 11 | 0.43 ± 0.05 | 9 | 0.9 ± 0.1 | 39 ± 4.1 |
| R111K | 0.06 ± 0.01 | 14 | 0.44 ± 0.04 | 11 | 1.0 ± 0.1 | 37 ± 3.1 |
| R111S | 0.17 ± 0.03 | 20 | 0.80 ± 0.06 | 8 | 0.8 ± 0.1 | 24 ± 3.9 |

**cAMP dependence**

| | $EC_{50}$ (μM) | H | n |
|---|---|---|---|
| WT # | 17 ± 0.77 | 3.1 ± 0.4 | >3 |
| Y26F | 40 ± 1.70 | 1.6 ± 0.1 | >3 |
| R120A | 37 ± 13.1 | 1.1 ± 0.4 | >4 |

Supplementary Table 1 summarizes numerical values obtained from analyzing single channel electrophysiology recordings obtained at 200 μM cAMP for the voltage dependence section of the table and at +100 mV for the cAMP dependence section of the table. Values are given as mean ± SEM. n is the number of experimental repeats. # indicates that the values (also at 200 μM cAMP) were taken from (Schmidpeter et al., 2018).

## Supplementary Table 2: Structure of SthK Y26F

| | SthK Y26F | |
|---|---|---|
| | Closed state | Activated state |
| PDB | 7RSH | 7RTJ |
| EMDB | 24670 | 24682 |
| **Data collection and processing** | | |
| Nominal magnification | 130000 | |
| Voltage (kV) | 300 | |
| Electron exposure (e-/Å$^2$) | 54.6 | |
| Defocus range (µm) | -1 to -3 | |
| Pixel size (Å) | 1.06 | |
| Symmetry imposed | C4 | C4 |
| Initial particle images (no.) | 1,324,724 | |
| Final particle images (no.) | 46,786 | 109,647 |
| Map resolution (Å) at FSC 0.143 | 3.0 | 3.8 |
| Map local resolution range (Å) | 2.9-4.7 | 3.7-5.9 |
| **Refinement** | | |
| Initial model used | 6CJU | 6CJU |
| Model resolution (Å) (at FSC 0.5) | 3.1 | 3.8 |
| Model resolution range (Å) | 2.5 – 3.1 | 3.1 – 3.8 |
| Map sharpening b-factor (Å$^2$) | -87 | -153 |
| Model composition | | |
| Nonhydrogen atoms | 13744 | 13352 |
| Protein residues | 1604 | 1592 |
| Ligands | PGW: 56<br>CMP: 4 | PGW: 32<br>CMP: 4 |
| B-factors | | |
| Protein | 7.49/82.01/35.82 | 1.95/80.64/46.85 |
| Ligands | 13.34/67.65/38.25 | 9.14/66.31/38.34 |
| RMSD | | |
| Bond length (Å) | 0.006 | 0.007 |
| Bond angle (°) | 1.139 | 1.020 |
| **Validation** | | |
| MolProbity score | 1.93 | 1.91 |
| Clash score | 8.09 | 10.58 |
| Poor rotamers (%) | 0.58 | 0 |
| Ramachandran plot | | |
| Favored (%) | 92.00 | 94.67 |
| Allowed (%) | 8.00 | 5.33 |
| outliers (%) | 0 | 0 |

Supplementary Table 2 provides data collection and structure validation details for SthK Y26F.

## Supplementary Table 3: Structure of SthK R120A

| | SthK R120A | | | |
|---|---|---|---|---|
| | Closed state | O1 | O2 | O3 |
| PDB | 7RTF | 7RU0 | 7RYS | 7RYR |
| EMDB | 24681 | 24692 | 24747 | 24746 |
| **Data collection and processing** | | | | |
| Nominal magnification | 81000 | | | |
| Voltage (kV) | 300 | | | |
| Electron exposure ($e^-/Å^2$) | 61.28 | | | |
| Defocus range (μm) | -1.5 to -2 | | | |
| Pixel size (Å) | 1.09 | | | |
| Symmetry imposed | C4 | C4 | C4 | C4 |
| Initial particle images (no.) | 3,004,702 | | | |
| Final particle images (no.) | 799,349 | 50,037 | 149,792 | 46,629 |
| Map resolution (Å) at FSC 0.143 | 2.9 | 4.22 | 3.59 | 3.64 |
| Map local resolution range (Å) | 2.6 – 4.0 | 3.9 – 5.7 | 3.4 – 5.0 | 3.4 – 5.2 |
| **Refinement** | | | | |
| Initial model used | 6CJU | 7RTJ | 7RU0 | 7RTJ |
| Model resolution (Å) (at FSC 0.5) | 2.9 | 6.5 | 3.9 | 3.7 |
| Model resolution range (Å) | 2.6 – 2.9 | 3.4-6.5 | 3.2-3.9 | 3.1-3.7 |
| Map sharpening b-factor ($Å^2$) | -102 | -218 | -171 | -123 |
| Model composition | | | | |
| Nonhydrogen atoms | 13460 | 12284 | 12900 | 13024 |
| Protein residues | 1580 | 1536 | 1556 | 1556 |
| Ligands | PGW: 48<br>CMP: 4 | CMP: 4 | PGW: 28<br>CMP: 4 | PGW: 28<br>CMP: 4 |
| B-factors | | | | |
| Protein | 0.00/72.09/<br>30.07 | 112.19/350.92/<br>231.38 | 0.20/47.30/<br>33.20 | 0.00/103.95/<br>55.46 |
| Ligands | 3.68/74.77/<br>39.94 | 252.94/252.94/<br>252.94 | 18.57/50.52/2<br>9.84 | 12.27/88.05/<br>52.20 |
| RMSD | | | | |
| Bond length (Å) | 0.004 | 0.004 | 0.006 | 0.006 |
| Bond angle (°) | 0.904 | 1.122 | 1.051 | 1.075 |
| **Validation** | | | | |
| MolProbity score | 1.73 | 1.89 | 1.75 | 1.84 |
| Clash score | 5.19 | 8.34 | 6.50 | 6.98 |
| Poor rotamers (%) | 0 | 0 | 0.30 | 0.60 |
| Ramachandran plot | | | | |
| Favored (%) | 92.84 | 93.12 | 94.26 | 92.95 |
| Allowed (%) | 7.16 | 6.88 | 5.74 | 7.05 |
| outliers (%) | 0 | 0 | 0 | 0 |

Supplementary Table 3 provides data collection and structure validation details for SthK R120A.

**Supplementary Table 4: Summary of sm-FRET data**

| | High-FRET | | Intermediate-FRET | | Low-FRET | | |
|---|---|---|---|---|---|---|---|
| **SthK -cAMP** | FRET | % Area of FRET state | FRET | % Area of FRET state | FRET | % Area of FRET state | n |
| **WT** | 0.94±0.01 | 51.4±3.5 | 0.81±0.01 | 48.6±3.0 | none | | 53 |
| **Y26F** | 0.94±0.01 | 53.4±7.2 | 0.80±0.01 | 46.6±7.5 | none | | 53 |
| **R120A** | 0.94±0.01 | 60.1±6.7 | 0.81±0.01 | 39.9±6.8 | none | | 63 |
| **SthK +cAMP** | | | | | | | |
| **WT** | 0.92±0.02 | **49.4±4.5** | 0.78±0.01 | **40.0±3.8** | 0.59±0.04 | **10.6±0.7** | 58 |
| **Y26F** | 0.90±0.02 | **34.7±3.1** | 0.78±0.01 | **48.4±3.1** | 0.58±0.02 | **16.9±0.1** | 79 |
| **R120A** | 0.90±0.03 | **30.9±5.0** | 0.77±0.01 | **53.0±4.6** | 0.59±0.02 | **16.1±0.4** | 73 |

Supplementary Table 4 summarizes the numerical values obtained from sm-FRET experiments as presented in Fig 4. Values represent mean ± S.E.

**Supplementary Table 5: Oligonucleotides used in this study**

| Mutation introduced | primer sequence |
|---|---|
| Y26A | 5' - 3' CGGTCACCCTCTACGCCGCGATCCGCATCC<br>3' - 5' GGATGCGGATCGCGGCGTAGAGGGTGACCG |
| Y26N | 5' - 3' CGGTCACCCTCTACAACGCGATCCGCATCC<br>3' - 5' GGATGCGGATCGCGTTGTAGAGGGTGACCG |
| Y26F | 5' - 3' CGGTCACCCTCTACTTCGCGATCCGCATCC<br>3' - 5' GGATGCGGATCGCGAAGTAGAGGGTGACCG |
| R120A | 5' - 3' CATCTCGGTGCAGGCGAGCGCCACCCGG<br>3' - 5' CCGGGTGGCGCTCGCCTGCACCGAGATG |
| L54F | 5' - 3' GAGCCTCGCCTTCATCGCCGACATCCCC<br>3' - 5' GGGGATGTCGGCGATGAAGGCGAGGCTC |
| L54W | 5' - 3' GAGCCTCGCCTGGATCGCCGACATCCCC<br>3' - 5' GGGGATGTCGGCGATCCAGGCGAGGCTC |
| L54Y | 5' - 3' GAGCCTCGCCTACATCGCCGACATCCCC<br>3' - 5' GGGGATGTCGGCGATGTAGGCGAGGCTC |
| R111K | 5' - 3' CTCTCGCTCGTCAAGCTCCTCAAGCTCATCTCG<br>3' - 5' CGAGATGAGCTTGAGGAGCTTGACGAGCGAGAG |
| R111S | 5' - 3' CTCTCGCTCGTCAGCCTCCTCAAGCTCATCTCG<br>3' - 5' CGAGATGAGCTTGAGGAGGCTGACGAGCGAGAG |
| R111Q | 5' - 3' CTCTCGCTCGTCCAGCTCCTCAAGCTCATCTCG<br>3' - 5' CGAGATGAGCTTGAGGAGCTGGACGAGCGAGAG |
| S51A | 5' - 3' GACATCCTCGCGGCCCTCGCCCTCATCGCC<br>3' - 5' GGCGATGAGGGCGAGGGCCGCGAGGATGTC |
| C153A | 5' - 3' CACGGCATCGCCGCCGGATGGATGAGCCTC<br>3' - 5' GAGGCTCATCCATCCGGCGGCGATGCCGTG |
| D57A | 5' - 3' GCCCTCATCGCCGCCATCCCCCTCGACCTTGCC<br>3' - 5' GGCAAGGTCGAGGGGGATGGCGGCGATGAGGGC |
| D57N | 5' - 3' GCCCTCATCGCCAACATCCCCCTCGACCTTGCC<br>3' - 5' GGCAAGGTCGAGGGGGATGTTGGCGATGAGGGC |
| D84A | 5' - 3' CGCCTTCCCGCCCTCCTGGCGGCACTC<br>3' - 5' GAGTGCCGCCAGGAGGGCGGGAAGGCG |
| D84N | 5' - 3' GCCTTCCCAACCTCCTGGCGGCACTC<br>3' - 5' GAGTGCCGCCAGGAGGTTGGGAAGGC |
| D92A | 5' - 3' CGGCACTCCCGCTCGCTCTCTTGGTGTTCGCC<br>3' - 5' GGCGAACACCAAGAGAGCGAGCGGGAGTGCCG |
| D92N | 5' - 3' CGGCACTCCCGCTCAACCTCTTGGTGTTCGCC<br>3' - 5' GGCGAACACCAAGAGGTTGAGCGGGAGTGCCG |

Supplementary Table 5 summarizes all mutational primers used in this study.

References:

Rheinberger, J., X. Gao, P.A. Schmidpeter, and C.M. Nimigean. 2018. Ligand discrimination and gating in cyclic nucleotide-gated ion channels from apo and partial agonist-bound cryo-EM structures. *Elife*. 7:e39775.

Schmidpeter, P.A.M., X. Gao, V. Uphadyay, J. Rheinberger, and C.M. Nimigean. 2018. Ligand binding and activation properties of the purified bacterial cyclic nucleotide-gated channel SthK. *J Gen Physiol*. 150:821-834.

Shuang, B., D. Cooper, J.N. Taylor, L. Kisley, J. Chen, W. Wang, C.B. Li, T. Komatsuzaki, and C.F. Landes. 2014. Fast Step Transition and State Identification (STaSI) for Discrete Single-Molecule Data Analysis. *J Phys Chem Lett*. 5:3157-3161.
